# Hepatoblastoma in an Adult with Biliary Obstruction and Associated Portal Venous Thrombosis

**DOI:** 10.1155/1995/16832

**Published:** 1995

**Authors:** Lav K. Kacker, E. M. Khan, Rohit Gupta, V. K. Kapoor, Rakesh Pandey, R. K. Gupta, V. A. Saraswat

**Affiliations:** 1 Department of Surgical Gastroenterology Sanjay Gandhi Postgraduate Institute of Medical Sciences; Raebareli Road Lucknow UP India; 2 Department of Pathology Sanjay Gandhi Postgraduate Institute of Medical Sciences; Raebareli Road Lucknow UP India; 3 Department of Gastroenterology Sanjay Gandhi Postgraduate Institute of Medical Sciences; Raebareli Road Lucknow UP India; 4 Department of Radiodiagnosis Sanjay Gandhi Postgraduate Institute of Medical Sciences; Raebareli Road Lucknow UP India

## Abstract

We present a case of adult hepatoblastoma. This young female presented with severe acute cholangitis. Preoperative diagnosis was common bile duct (CBD) obstruction with portal vein thrombosis. On exploration she had a tumor mass in the CBD. The unusual features of this case are discussed in this report.


*HPB Surgery*, 1995, Vol. 9, pp. 47–49
Reprints available directly from the publisher
Photocopying permitted by license only

© 1995 OPA (Overseas Publishers Association)
Amsterdam B.V. Published in The Netherlands
by Harwood Academic Publishers GmbH
Printed in Malaysia

CASE REPORT

### Hepatoblastoma in an Adult with Biliary Obstruction and Associated Portal Venous Thrombosis

LAV K. KACKER*, E. M. KHAN**, ROHIT GUPTA***, V. K. KAPOOR*, RAKESH PANDEY**, R. K. GUPTA#, and V. A. SARASWAT***,

*Department of Surgical Gastroenterology,**Department of Pathology,***Department of Gastroenterology, #Department of Radiodiagnosis, Sanjay Gandhi Postgraduate Institute of Medical Sciences; Raebareli Road, Lucknow UP, India

*(Received 23 May 1993)*

We present a case of adult hepatoblastoma. This young female presented with severe acute cholangitis. Preoperative diagnosis was common bile duct (CBD) obstruction with portal vein thrombosis. On exploration she had a tumor mass in the CBD. The unusual features of this case are discussed in this report.

KEY WORDS: Hepatoblastoma portal vein thrombosis biliary obstruction

### INTRODUCTION

Hepatoblastomas are tumors of children and are rare in adults. We report a case of hepatoblastoma in a 28 year old woman with unusual clinical, morphologic and histologic features.

### CASE REPORT

A 28 year old woman presented with presistent, epigastric and right upper quadrant pain, cholestatic jaundice and intermittent, low grade fever for 2 months. Examination revealed anaemia, deep jaundice and pedal edema. Her temperature was 38.4°C. The liver was palpable 4 cm below the right costal margin and was tender and smooth. Free fluid was present in the abdomen. The gall bladder and spleen were not palpable. Hemoglobin was 8.2 gm%; WBC count 6000 cells/cumm with 80% polymorphs; blood sugar and renal function were within normal limits; total serum protein was 4.8 gm% and serum albumin 2.1 gm%; total serum bilirubin 20. 8 mg% with direct bilirubin 12.0 mg%; alkaline phosphatase 166 U/L (range 35–170 U/L); SGOT and SGPT 70 and 12 U/L (range 5–40 U/L) respectively. Prothrombin time and activated partial thromboplastin time were marginally prolonged. The ascitic fluid was transudative and exfoliative cytology was negative for malignant cells. Upper gastro-intestinal endoscopy did not reveal oesophagogastric varices. Ultrasonographic examination showed an irregular liver surface with loss of normal architecture. The gall bladder was markedly distended with sludge and the CBD was dilated down to the the lower end with the echogenicity of the bile similar to that of the gall bladder, suggesting sludge in the CBD. Intrahepatic biliary ducts were dilated in the left lobe while right lobe ducts could not be visualized. There was no space occupying lesion in the liver. There was a thrombus in the extrahepatic portal vein with periportal and retro-peritoneal collaterals.

Percutaneous liver biopsy, done at this stage, did not show any evidence of liver cirrhosis. However,

*Address for correspondence:* VA Saraswat, Associate Professor, Department of Gastroenterology, Sanjay Gandhi Postgraduate Institute of Medical Sciences, Raibareli Road, Lucknow, India.

cholestasis, mild bile ductular proliferation and polymorphonuclear infiltration suggested extrahepatic bile duct obstruction with cholangitis. In order to obtain a better delineation of the anatomy, a CT scan was performed, which confirmed the sonographic findings and showed that CT attenuation values of gall bladder and CBD contents were similar (36 HU). ERCP showed gross dilatation of the CBD (20 mm) with a large intra-luminal filling defect (4.5 x 1.5 cm). During injection, contrast could be seen streaming around this mass which extended from just above the papilla to the porta hepatis. The right hepatic duct and its branches were blocked while the left hepatic duct was opacified adequately.

To relieve the cholangitis, an endoscopic nasobiliary drain was placed in the left hepatic duct. This drained about 50 ml bile per day and bile culture grew Pseudomonas aeruginosa. Despite appropriate antibiotic therapy and nasobiliary drainage, cholangitis continued and operative decompression of the biliary system was necessary. The preoperative diagnosis was liver cirrhosis, probably cryptogenic, with portal hypertension, portal vein thrombosis, extrahepatic biliary obstruction due to CBD sluge or stones and liver failure. Exploration revealed macronodularity of both lobes of the liver and ascites. The gall bladder was distended with sludge and thick viscid bile. Dilated vessels were present along the hepatoduodenal ligament. The CBD was grossly dilated (3cm) and filled with fleshy and necrotic tissue which was removed after choledochotomy. Extra hepatic biliary ducts were cleared and a T-tube was placed. Cholecystostomy was also done. Needle biopsies of the liver were taken. During the postoperative period the cholangitis settled. T-tube cholangiogram on the 14th postoperative day showed a blocked right hepatic duct. The left ductal system and CBD were dilated but free of any filling defects and constrast flowed freely into the duodenum. The patient was discharged after 2 weeks with the T-tube in situ.

Liver biopsies obtained during surgery showed extensive parenchymal and canalicular cholestasis and features of acute cholangiohepatitis. There was no evidence of liver cirrhosis and no tumor tissue was seen in the biopsy. Tissue obtained from the CBD consisted of multiple small, soft, fleshy and necrotic pieces. Microscopically, the tumor was predominantly composed of sheets, trabecular cords and islands of polyhedral cells separated by sinusoidal channels lined by endothelial cells. These cells showed slightly anisomorphic, round to oval nuclei with inconspicuous nucleoli and abundant pale to lightly granular cytoplasm. At places, islands of small, primitive, embryonal cells were present with hyperchromatic nuclei and a narrow rim of eosinophilic cytoplasm. The mesenchymal component was seen as irregular bands and large islands of primitive fibro-myxomatous appearance with plump, spindle shaped and elongated nuclei. Frequent mitotic figures (1-3 HPF) were seen. Many of the fragments were completely necrotic. Histopathologic diagnosis was hepato-blastoma with cholestasis and cholangiohepatitis.

Liver function parameters 4 weeks after surgery did not show any improvement. Serum bilirubin was 20 mg% with direct fraction of 8.9 mg%; serum alkaline phosphatase 132 U/L; SGOT/SGPT 59 and 124 U/L respectively, total proteins 5.6 gm% with serum albumin 2.0 gm%. Serum alfafetoprotein (AFP) level was 9000 IU/ml (normal 0.5–55 IU/ml). The patient was lost to followup after this hospital visit.

### DISCUSSION

This 28 year old woman with hepatoblastoma involving the intrahepatic and extrahepatic biliary channels presented with several unusual features.

Hepatoblastoma is very rare in adults. It is primarily a tumor of young children with 92% of the cases presenting below the age of 5 years[1]. Only 20 cases of adult hepatoblastoma have been reported in the English literature[2–8]. Jaundice, as a presenting feature, is uncommon in hepatoblastoma having been noted in less than 6% of cases[9]. This is the first report of adult hepatoblastoma presenting with obstructive jaundice. A variety of mechanisms are responsible for jaundice in liver tumors[10]. These include tumor infiltration into the hepatic parenchyma, compression of biliary channels by tumor mass or lymphnodes, tumor necrosis with hemobilia, pedunculated tumor extension, clot or tumor debris within the bile ducts and associated cirrhosis. In all likelihood, extension of the tumor into the right hepatic duct and the common bile duct from a microscopic parenchymal focus in the right lobe resulted in biliary obstruction and jaundice in our patient. The cholangiographic appearance of tumor invading the bile ducts is described as a smooth or lobulated intraluminal filling defect, giving rise to the'goblet' or 'wineglass' appearance[10]. This is identical to the appearance noted in our patient. Peroperatively tumors are described as fleshy, soft and compressible, similar to chicken fat[11], as was observed in our patient.

Hepatoblastoma commonly forms a single mass within the right lobe of the liver[1,2]; less commonly multiple nodules may be present, usually in both lobes[1,12].

The least common is diffuse tumor involving the entire liver. Cirrhosis is rarely present with hepatoblastoma[1,2]. In our patient no mass lesion was detected in the liver on US, CT or at surgery. Diffuse involvement of the liver was ruled out by negative liver biopsies. This unusual presentation of hepatoblastoma with a microscopic parenchymal focus growing primarily into the extrahepatic biliary ducts has not been reported before. Possibly, extension of tumor thrombus into the portal vein may have resulted in portal vein thrombosis, contributing to portal hypertension. The association of this complication with hepatoblastoma has also not been reported in the literature. Histologically, hepatoblastoma is classified into epithelial and mixed (epithelial and mesenchyml) types. The epithelial component may be of fetal or embryonal cell type[12]. There is a single case report of epithelial hepatoblastoma in an adult[4]. All other cases of adult hepatoblastoma are of mixed type, as was seen in our case.

### CONCLUSION

We report a case of hepatoblastoma in a 28 year old woman. The tumor had invaded the biliary channels and CBD causing jaundice and cholangitis. Portal vein thrombosis was also present and liver failure had developed probably due to the combination of biliary and portal venous obstruction along with associated severe cholangitis.

### REFERENCES

1. Stocker, J. T., Ishak, K. G. (1987) Hepatoblastoma. In : Okuda K, Ishak K. G., (Eds). *Neoplasms of the liver*. Springer Verlag, 127–36.
2. Honon, R. P., Haqqani, M. T., (1980) Mixed hepato-blastoma in the adult: case report and review of literature. *J. Clin. Pathol.*, **33**, 1050–63.
3. Yoshida, T., Okozaki, M., Yoshino, N., Shimamura, Y., Miyazawa, N., Miyamoto, K., Kishi, K. (1979) A case of hepatoblastoma in adult. *Jpn. J. Clin. Oncol.*, **9**, 163–8.
4. Bhatnagar, A., Pathania, O. P., Champakam, N. S. (1987) Hepatoblastoma in an adult female (letter). *Indian. J. Gastroenterol.*, **6**, 125–6.
5. Misra, G. C., Mansharamani G. G., Deewan, R. Jayaraman, G. (1988) Mixed hepatoblastoma in a young adult. *J. Assoc. Phys. India.*, **36**, 169–70.
6. Sugino, K., Dohi, K., Matsuyama, T., Asahara, T., Yamamoto, M. (1989) A case of hepatoblastoma occuring in an adult. *Jpn. J. Surg.*, **19**, 489–93.
7. Seabrook, G. R., Collin J. R., Britton, B. J. (1989) Hepatoblastoma : Successful resection in an adult. *Br. J. Clin. Pract.*, **43**, 345–6.
8. Oda, H., Honda, K., Hara, M., Arase, Y., Ikeda, K., Kumada, H. (1990) Hepatoblastoma in an 82 year old man. An autopsy case report. *Acta. Pathol. Jpn.*, **40**, 212–8.
9. Rosenberg, G. J. (1977) Hepatoblastoma-case report and literature review. *Clin. Proc. Child. Hosp. Nat. Med. Center.*, **33**, 11–9.
10. van Sonnenberg, E., Ferruci, J. T. (1979) Bile duct obstruction in hepatocellular carcinoma (Hepatoma)-Clinical and cholangiographic characterstics. Report of 6 cases and review of the literature. *Radiology*, **130**, 7–13.
11. Edmondson, H. A., Steiner, P. E. (1954) Primary carcinoma of the liver. A Study of 100 cases among 68900 necropsies. *Cancer*, **7**, 462–503.

